# Safety and Effectiveness of G-Mesh^®^ Gynecological Meshes Intended for Surgical Treatment of Pelvic Organ Prolapse—A Retrospective Analysis

**DOI:** 10.3390/jcm13237421

**Published:** 2024-12-05

**Authors:** Maciej Wilczak, Karolina Chmaj-Wierzchowska, Mariusz Wójtowicz, Przemysław Kądziołka, Paulina Paul, Aleksandra Gajdzicka, Kaja Jezierska, Witold Sujka

**Affiliations:** 1 Department of Maternal and Child Health and Minimally Invasive Surgery, Poznan University of Medical Sciences, 60-701 Poznan, Poland; 2 Municipal Hospital in Zabrze, Zamkowa 4, 41-803 Zabrze, Poland; 3 UROFEM Estetica Specialist Medical Practice, Kanclerska 2, 60-327 Poznan, Poland; 4 Tricomed S.A., Świętojańska 5/9, 93-493 Lodz, Poland

**Keywords:** pelvic organ prolapse (POP), laparoscopic surgery, Dubuisson, vaginal approach, laparotomy, gynecological mesh

## Abstract

**Background:** The prevalence of POP in women ranges from 30–40%, with 10–20% requiring surgical intervention. Annually, over 225,000 surgical procedures for POP are performed in the United States. The severity of prolapse is assessed using the four-stage POP-Q system, which facilitates clinical research by providing a standardized measure of defect severity. Surgical intervention is indicated for more severe cases, with various techniques available through vaginal or abdominal access. Synthetic meshes, primarily made of polypropylene (PP), are commonly used in POP surgeries due to their biocompatibility and mechanical support. This research aims to evaluate the effectiveness and safety of a non-resorbable, light polypropylene gynecological mesh (G-Mesh^®^, Tricomed S.A., Łódź, Poland) in the surgical treatment of pelvic floor prolapse in women. **Methods:** The meshes were implanted via laparoscopy (Dubuisson method) and laparotomy or transvaginally. A multicenter, retrospective study was conducted involving 81 patients aged 28–83. **Results:** The results collected at three follow-up visits indicated a high level of patient satisfaction, minimal discomfort, and no significant pain. Many patients emphasized significant improvement in quality of life and the lack of any adverse events associated with the presence of the implant. **Conclusions:** The G-Mesh^®^ gynecological mesh has emerged as an effective and safe intervention for treating pelvic floor dysfunction in women, addressing conditions such as cystocele, rectocele, uterine prolapse, and ureterocele.

## 1. Introduction

Pelvic floor muscles (PFM) consist of various structures that provide specific support for internal organs in women [1]. One of the effects of the weakening of the pelvic floor muscles is the disruption of the statics of the reproductive organs with their prolapse (POP—pelvic organ prolapse). It may affect both the anterior vaginal wall (cystocele—bladder prolapse) and posterior vaginal wall (rectocele—rectal prolapse), vaginal vaults, and uterus, but also the utero-rectal cavity (enterocele—prolapse of the small intestine into the vagina) and the urethra (ureterocele) [2]. The incidence of this disease in women is equal to 30–40%, and in 10–20% of cases surgery is required [3]. According to the literature data, over 225,000 procedures of this type are performed annually in the United States [4]. The level of prolapse of the pelvic organs, urinary bladder, and rectum is determined using the four-stage POP-Q system (Pelvic Organ Prolapse Quantification System), where the last, fourth degree means complete prolapse into the vagina. This scale is often used in scientific research because it describes the severity of a particular defect. When deciding the surgery type, the clinical division according to DeLancey is used more often, and it classifies three levels of damage to the lesser pelvis [5]. Risk factors increasing the occurrence of mentioned disorders include congenital connective tissue defects, obesity, and multiple, prolonged deliveries [6,7]. Pelvic organ prolapse is also often associated with urinary incontinence, including increased stress urinary incontinence (SUI) [8,9] or residual urine due to underbladder obstruction. Approximately 55% of women with stage 2 POP (hymenal prolapse ± 1 cm) have concomitant SUI, and only 33% of women with stage 4 POP have SUI [10].

The treatment of static disorders should be performed depending on the stage of the disease, reported symptoms, and the age of the patient. In young patients, in premenopausal patients regardless of the degree of pelvic organ prolapse, and in postmenopausal patients with a mild degree of prolapse of the reproductive organs and urinary bladder or intestine, conservative treatment is recommended, consisting of pessary therapy.

Depending on the severity of the prolapse of the reproductive organ, bladder, or rectum and the reported symptoms (pain and discomfort in the perineum, groin, sacrolumbar region, urinary urgency, frequent urination, difficulty emptying the bladder, constipation, or nocturia), the patient is qualified for pelvic floor reconstruction surgery [11]. Various surgical methods are used via vaginal or abdominal access (laparoscopic and laparotomy). Isolated defects at levels 1 and 2 according to DeLancey constitute an indication for sacropexy—suspension of the lowered/prolapsed organ to the sacrum (the preferred place for tape fixation is the S2–S3 height on the sacrum, as it is a safer place, associated with fewer complications compared to fixation at the promontorium level, and also allows for maintaining the physiological axis of the vagina) [12].

Laparoscopic lateral uteropexy using Dubuissone’s method may be an alternative to sacropexy also in obese patients [13]. During the operation, the urinary bladder is mobilized, and the anterior vaginal wall, together with the cervix, is suspended using a mesh fixed to the fascia of the external oblique muscle of the abdomen [14,15,16]. A modified operation can also be performed in patients after hysterectomy when, after mobilization of the urinary bladder and rectum, the vaginal apex is suspended using the tension-free suspension concept [17]. If there are contraindications to abdominal surgery, suspension to the sacrospinous ligament via a vaginal approach should be considered. In a level 2 defect (DeLancey level 2) for cystocele with a lateral defect, anterior gynecological mesh implantation is recommended, except for premenopausal patients, in whom laparoscopic colposuspension via Richardson’s surgery (paravaginal repair) is the preferred method. In the case of rectocele, posterior surgery is the method of choice, and in case of recurrence, posterior gynecological mesh implantation is possible [12]. However, the use of transvaginal mesh instead of native tissue during POP surgery has been shown to provide better anatomical results [18,19].

Most synthetic meshes used in POP surgeries are made of polypropylene (PP), a polymer well known to induce an early inflammatory response followed by a fibrotic response [20]. Gynecological mesh should have appropriate biological and mechanical properties. This means that the material used to create the implant must be biocompatible with the tissues but also should be both stiff and flexible in order to provide sufficient mechanical support [21]. Due to their weight, macroporous meshes are divided into three categories: high weight (HW) (density greater than 80 g/m^2^), medium weight (MW) (between 50 and 80 g/m^2^), or low weight (LW)—less than 50 g/m^2^ [22]. The high weight of the mesh reduces the porosity of the fibers, improving the durability of the material, but thus increasing the discomfort of the patient and the possibility of scarring, which significantly hinders the integration of the mesh with the tissues. This also affects the occurrence of chronic pain [23]. The development of a light mesh with a larger pore size and less polypropylene material allows for the reduction of negative effects [24].

The aim of the conducted research was to evaluate the effectiveness and safety of a non-resorbable, light, polypropylene gynecological mesh (G-Mesh^®^, Tricomed S.A., Poland) for the surgical treatment of pelvic floor prolapse in women.

## 2. Materials and Methods

### 2.1. Description of the Procedure 

A multicenter, retrospective study was conducted involving 81 patients undergoing surgical treatment for pelvic organ prolapse. The study was based on patient data obtained from medical records created on the basis of routinely performed surgical procedures reimbursed by the Polish National Health Fund (NFZ), during which gynecological meshes were implanted. The surgeries took place between 2019 and 2023. The meshes were implanted via transabdominal access by laparoscopy and laparotomy or transvaginally. The procedures were performed by qualified surgeons in three different centers in Poland (Center 1—Karol Marcinkowski Medical University in Poznan, Department of Maternal and Child Health, Maternal and Child Health Clinic in Poznan; Center 2—Urofem Estetica Specialist Medical Practice in Poznan; Center 3—Municipal Hospital in Zabrze).

The study only included patients for whom full medical records were available, collected during at least one follow-up visit after the implantation of the G-Mesh^®^ (Tricomed S.A., Łódź, Poland) gynecological mesh. Demographic, preoperative, operative, and postoperative information was obtained from the hospital database. The study included clinical classification according to DeLancey and the Pelvic Organ Prolapse Quantification System (POP-Q). Based on the above data and descriptions of the type of pelvic floor static disorders, a CRF (case report form) was completed for each patient. After diagnosis and surgery, perioperative data were collected, including the gynecological mesh implantation technique used, the type of device used, the duration of surgery, intraoperative complications, and adverse events related to the implant.

Early postoperative assessment included the patients’ subjective feelings and clinical aspects, such as the duration of hospitalization, implementation and course of antibiotic therapy during hospitalization, and description of complications and adverse events related to the implant that occurred in the first few days after surgery. Additionally, the operating surgeon/gynecologist conducted a detailed interview with the patients, during which the comfort or discomfort felt after the procedure was determined. Postoperative pain sensation was assessed using a visual analog scale (VAS). Functional assessment was also performed to determine the impact of mesh implantation on the patients’ quality of life.

During the first follow-up visit after gynecological mesh implantation, which was an inclusion criterion for the study, as well as during the early post-surgery assessment, the postoperative complications and adverse events related to gynecological mesh implantation were checked. The patients’ comfort, pain sensation, and physical function after the procedure were evaluated. Additionally, the effectiveness and safety of the product used were also assessed. The subjective feeling of the presence of a foreign body after the procedure was also checked. For approximately half of the patients, similar data were also collected from the second follow-up visit.

As part of an additional visit, all patients were interviewed by telephone. During this, conversations about their comfort related to the implant were assessed, and postoperative pain was also determined.

Ethical review and approval were waived for this study because the scientific study conducted did not have the characteristics of a medical experiment and, in accordance with Polish law and Good Clinical Practice (GCP), was not subject to the opinion of the Bioethics Committee.

### 2.2. Patient Population

The clinical study included 81 patients aged 28–83 years who were qualified for treatment of pelvic floor organ prolapse using G-Mesh^®^ gynecological mesh and for whom complete medical documentation was available, collected during at least one follow-up visit approximately 3 months after the procedure. The following exclusion criteria were adopted: pregnancy, intake of immunosuppressive drugs and steroids, liver cirrhosis, infections, thrombocytopenia (platelet count < 100 × 10^9^/L), chronic renal failure, and patients diagnosed with mental illness. 

The patients were divided into two groups. The first group consisted of patients who had a gynecological mesh implanted laparoscopically, using the Dubuisson method (*n* = 66), and the second group consisted of patients who had a gynecological mesh implanted via other surgical techniques, i.e., transvaginally (*n* = 13) and using the Richardson method in laparotomy (*n* = 2). Transvaginal methods and laparotomy were less preferred by surgeons performing the surgery, as they are associated with a higher risk of perioperative and postoperative complications compared to laparoscopy. Due to the ongoing controversy regarding the effectiveness of these methods, the paper included a discussion and an attempt to compare them with laparoscopic methods based on available data.

### 2.3. Description of the Product

G-Mesh^®^ gynecological mesh is used in surgical treatment of the lowering of the reproductive organs in women. It is a medical device intended for reconstructive techniques, i.e., reproducing the normal anatomy of the pelvis, providing support for internal organs such as the urinary bladder, uterus/vaginal vault, or rectum.

G-Mesh^®^ gynecological mesh with a macroporous structure is made of polypropylene, a monofilament yarn with a fiber thickness of 0.10 mm in transparent and blue. The used raw material has a high breaking strength (430 ÷ 540 cN), which ensures a medical product with the assumed strength parameters. The surface weight of the knitted fabric of the finished product is 40 g/m^2^ (with a tolerance of ±5). The surface area of the macropores is max. 1.4 mm^2^, while the porosity of the mesh is about 68%. The raw material is obtained from 100% polypropylene homopolymer. Additionally, the ends of the mesh arms are equipped with protrusions that make it easier to insert the arms through the holes in the applicator. The dimensions of the G-Mesh^®^ product are presented in Table 1. The product does not contain any substances that, when used separately, may be considered a medicinal product, nor does it contain any blood products or non-viable tissues of animal origin. The product is not obtained from tissues or products of human origin. G-Mesh^®^ gynecological mesh is characterized by low surface weight, atraumatic edges, a stable knitted structure, high elasticity, high stability, a lack of irritating and allergenic effects, and a lack of toxicity, cytotoxicity, or genotoxicity. Table 1 presents the division of meshes depending on the span, length, and width of the arms.

**Figure 1:**
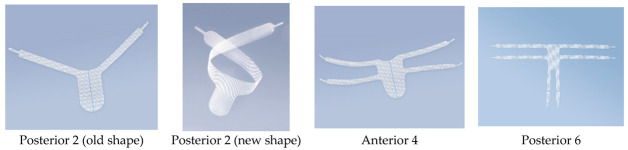
G-Mesh^®^ gynecological mesh.

G-Mesh gynecological mesh is available in three variants (Figure 1): G-Mesh^®^ Posterior 2 (two-armed, two versions), Anterior 4 (four-armed), and Posterior 6 (six-armed). The decision regarding the implantation method is made by a specialist, but it is recommended that patients with various symptoms should be treated as follows: rectocele or enterocele should be treated with Posterior 2 or Posterior 6; cystocele should be supplied with Anterior 4; and lowering of the uterus or vaginal vault after hysterectomy should be treated with the Posterior 6 mesh. Depending on the type of G-Mesh^®^, the mesh is recommended to be attached as follows:

- Posterior 2—Make a bilateral incision in the skin in the buttock area. Place the implant in the posterior vaginal wall from the rectum side, with the arms facing upwards, from the sacrospinous ligament. Attach the arms from the sacrospinous ligament and guide their ends through the tissues towards the skin incisions.
- Anterior 4—Make two bilateral incisions in the skin in the groin (upper and lower). Place the implant in the anterior vaginal wall from the bladder side, with the arms facing the obturated openings and with the narrower part (tongue) towards the vaginal opening. Pull the arms through the anterior part and the posterior angle of the obturated openings and guide their ends through the tissues towards the skin incisions. Make the first incision in the area of the genital-femoral line and the second incision 3 cm lower and 2 cm lateral to the first incision.
- Posterior 6—It is possible to cut off the two lateral arms. Make three or two incisions bilaterally (for six or four arms). Make the first incision in the genital-femoral area and pass the first one from the narrower side of the mesh through the anterior part of the obturator holes. Pull the end of the arm through the tissues towards the incisions in the skin. Make the second incision 3 cm lower and 2 cm lateral to the first incision. Pull the second (perpendicular) arm to the second incision through the posterior angle of the obturator hole. Place the implant in the posterior vaginal wall from the rectum side, with the narrower part towards the vaginal opening. Attach the remaining two arms to the sacrospinous ligament and pass through the third opening located in the gluteal area.

### 2.4. Implantation Technique

Mesh implantation procedures were performed using three methods: laparoscopically using the Dubuisson method, transvaginally, and using Richardson’s method in laparotomy.

**Figure 2:**
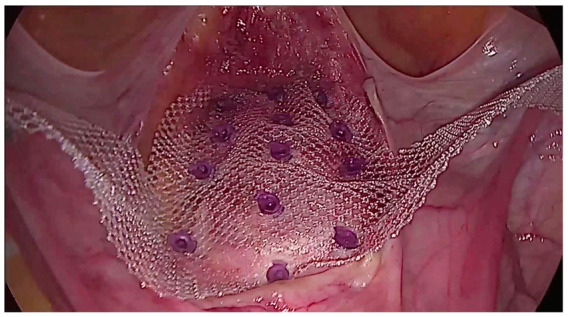
View of the G-Mesh^®^ two-arm mesh partially attached to the cervix, vaginal apex, and anterior vaginal wall using soluble tackers.

**Figure 3:**
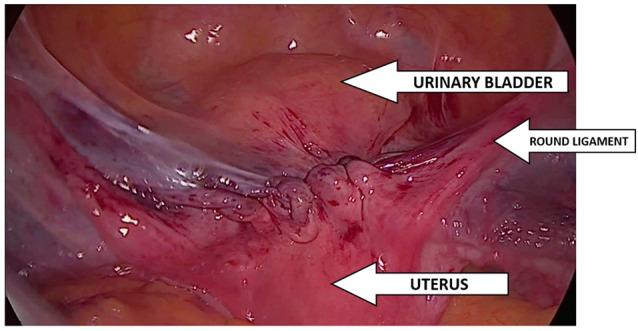
Final result of implantation of two-armed G-Mesh^®.^

Dubuisson’s laparoscopic lateral uteropexy using mesh was first reported for the treatment of genital prolapse by Dubuisson in 1998. Several modifications of the procedure have been described, but it is currently performed according to the standard surgical procedure, which has not been modified since 2003 [25]. The Dubuisson surgery is performed in the Trendelenburg position at an angle of approximately 15–30°, which ensures optimal visualization of the vesico-uterine space. The procedure begins with precise dissection of the vesico-uterine junction, dissection of the bladder, and its mobilization, which is crucial for the further stages of the operation. Then, a polypropylene mesh is introduced, shaped, and carefully placed over the front wall of the vagina and the cervix. The mesh is attached to the vesicouterine fascia using tackers and insoluble sutures, which ensures stable and durable support [26]. Figure 2 shows the prepared vesicovaginal space with the mesh attached. The effect of the operation after implanting the mesh, tightening the arms, and suturing the peritoneum with a continuous suture is shown in Figure 3.

In cases where laparoscopic lateral suspension is performed in post-hysterectomy patients, the technique involves suturing the mesh to the apex of the vagina using non-dissolvable sutures and tackers. The mesh is attached to both the front and back vaginal walls, which increases the stability and effectiveness of the treatment. After completing the main stages of the operation, the vesicoureteral fold of the peritoneum is carefully sutured. This step is intended to reduce tension in the peritoneal cavity, which is important for the patient’s comfort and recovery. Finally, the arms of the mesh are trimmed at the level of the skin, completing the procedure. This approach to surgery using the Dubuisson method allows for effective support of the pelvic structures, contributing to improved clinical results and patient comfort after surgery [25].

Transvaginal mesh implantation involves making an incision in the anterior (DeLancey level 2 defect, in the anterior compartment) or posterior (DeLancey level 2 defect, in the posterior compartment) vaginal wall and dissecting the tissue along its entire thickness, thus creating space for the implant. In the case of anterior mesh implantation, the bladder should be dissected (up to the vesicovaginal fascia) and all the way to bilateral access behind the obturator foramen and to the ischial spine. After localization of the sacrospinous ligament, the ligament should be dissected from the surrounding structures. In the case of posterior mesh implantation, the rectum should be dissected (to the rectovaginal fascia) up to bilateral access to the ischial spine, along with preparation of the sacrospinous ligament [13]. The arms of the mesh should be passed through the skin incisions, the excess cut off, and disposed of. The surgery is completed by closing the skin incisions with sutures. During the procedure, the patient’s anatomy should be carefully assessed, and care should be taken when passing the mesh through tissue structures, paying particular attention to large blood vessels, nerves, the bladder, and intestines. The placement and fixation of the implant depend on the type of mesh used.

Richardson’s procedure begins with a Pfannenstiel incision, which allows the surgeon to access the obturator space in the midline, continuing the dissection laterally, and completely separating the bladder from the anterior pubic ramus and urethra. The dissection is carefully continued inferiorly until the upper 2 to 3 cm of the medial attachment of the levator ani muscle is visible. At this point, a defect in the lateral pubocervical fascia can be identified. The medial edge of the separation usually retracts almost to the lateral superior vaginal groove. The surgeon elevates the superior lateral vaginal groove and places a mesh through the fascia adjacent to this groove and at the levator ani attachment, securing it with sutures to the superior pubic ramus.

### 2.5. Statistical Analysis

Clinical results and patient characteristics were expressed as mean ± SD (percentage). A Grubbs test was used to test statistically significant data, with the significance level set at *p* < 0.05.

## 3. Results

### 3.1. Demography

Our study included 81 patients from 3 centers. Patient demographics and preoperative statuses are presented in Table 2.

Before surgery, all patients underwent diagnostic tests. Four pelvic floor static disorders were diagnosed: lowering of the anterior vaginal wall/urinary bladder (Cystocele), lowering or prolapse of the uterus (Uterine prolapse), lowering of the vaginal vault/urethra (Ureterocele), and lowering of the posterior vaginal/rectal wall (Rectocele). A gynecological examination was performed, the level of depression of the pelvic organs and the urinary bladder was determined using the four-stage POP-Q system, and a cough test was performed. Preoperative data are summarized in Table 3.

All patients underwent surgical treatment of pelvic organ prolapse with implantation of gynecological mesh (Table 4). Only two-arm meshes were implanted using the Dubuisson method (laparoscopic access), whereas all three types of meshes were implanted using other methods. G-Mesh^®^ Posterior 2 mesh (two-arm) was used in 68 patients, Anterior 4 mesh (four-arm) was used in 2 patients, and Posterior 6 mesh (six-arm) was used in 11 patients. The mean operating time for all patients was 83 min. No adverse events related to the implant were recorded. During the operation, intraoperative complications such as postoperative wound tightening and difficulty in dissecting the bladder from the vagina were noted in only three patients.

### 3.2. Post-Operative Information

All patients stayed in the hospital for an average of 3 days after the surgery. During this time, complications related to the surgery were noted in only two patients (recurrent cystocele/rectocele and pain at the laparoscopic puncture site). No implant-related adverse events occurred. All postoperative information is summarized in Table 5.

The feeling of comfort/discomfort related to the implanted mesh was also assessed. None of the patients experienced any discomfort related to the implant. Virtually all patients (98%) rated the comfort as very good. Sense of function/dysfunction was also assessed. As many as 75 patients rated it as good, while 3 patients rated it as very good.

The VAS scale (0–10 scale) was used to assess pain after the procedure. In 79 patients, the pain sensation value was “0”—no pain (patients operated on using other techniques). In the remaining patients, the pain sensation value was “2” (two patients operated on laparoscopically and one patient operated on using another technique) or “3” (one patient operated on using another technique).

### 3.3. Follow up (Control Visit + Optional Visit)

The first follow-up visit took place on average 76 days after the G-Mesh^®^ implantation procedure (79 days in patients after laparoscopic surgery, 63 days in patients after surgery using another surgical technique). All patients (*n* = 81) attended the follow-up visit, which allowed for a complete assessment of the patients’ condition (Table 6). No adverse events directly related to the use of the G-Mesh^®^ device were observed. Additionally, no postoperative complications were recorded in 33 patients (30 patients after laparoscopic surgery, 3 patients after surgery using another method).

No foreign body sensation was reported in 66 patients, while 8 patients reported implant sensation (7 after laparoscopic surgery and 1 in the other group). No information on this subject was obtained from the remaining 7 patients.

The effectiveness and safety of the G-Mesh^®^ implant were assessed in most cases as positive and at a high level. Comfort after the procedure was also rated as very good or good, except for three patients who rated it as low (after laparoscopic surgery). Most patients did not experience postoperative pain at the first follow-up visit (58 patients after laparoscopic surgery, 15 patients from the other group).

An additional follow-up visit was optional and was attended by 42 patients (38 patients after laparoscopic surgery and 4 patients from the second group). The data collected at the second follow-up visit are presented in Table 7. The average time after which the visit took place was 178 days. No post-operative complications were reported in 16 patients, while their occurrence was noted in 26 patients.

No adverse events related to the use of the G-Mesh^®^ product were reported by 39 patients; 2 patients experienced a recurrence of the disease; 1 patient did not respond. Foreign body sensation was reported by 10 patients, and the absence of foreign body sensation was noted in 29 patients; 3 patients did not provide information on this subject.

During an additional visit, the effectiveness and safety of the G-Mesh^®^ implant were again assessed positively and at a satisfactory level. Comfort after the procedure was rated as very good or good by most patients.

In addition to follow-up visits, a detailed interview with the patients was also conducted, which took place on average 318 days after the procedure (Table 8). An interview was conducted with all patients (*n* = 81) and was aimed at assessing the subjective feeling of comfort and the level of pain after G-Mesh^®^ mesh implantation.

During the interview, the patients assessed the comfort after the procedure as very good. Many of them emphasized a significant improvement in the quality of life and the absence of any ailments related to the presence of the implant.

Pain sensation was measured using a visual analog scale (VAS) as on previous visits. The results showed that the average pain score was very low, indicating a complete absence of pain sensation, which further confirms the high effectiveness and tolerability of the G-Mesh^®^ implant.

## 4. Discussion

The introduction of minimally invasive gynecological surgery resulted in a significant increase in the number of advanced laparoscopic surgeries performed. This is mainly related to a number of advantages, including a reduced number of postoperative complications, the low invasiveness of the procedure, shortened hospitalization and recovery times, and better cosmetic results (less scarring). Additionally, the risk of postoperative complications is much lower than in the case of traditional surgical methods, e.g., surgery via abdominal (laparotomy) or transvaginal access [26,27,28]. A clinical study by Maher et al. (2011) [29] compared the effectiveness of the surgical treatment of pelvic floor lowering using laparoscopic and transvaginal techniques. The results of the study indicated that laparoscopic surgery resulted in a higher level of treatment satisfaction and greater effectiveness and was also characterized by lower morbidity and a lower risk of requiring reoperation compared to the transvaginal method. However, a study conducted in 2017 by To et al. [30] on a group of 413 women indicated that transvaginal mesh implantation may be as effective and safe as laparoscopic procedures. An important observation was also made by Withagen et al. (2013) [31]. Both methods proved to be comparably effective, but there are specific indications that influence the choice of the appropriate implantation technique, including age, previous surgeries, or the sexual activity of patients.

In 2008, the U.S. Food and Drug Administration (FDA) published the first warnings, with subsequent ones in 2011 and 2016, regarding the potential for serious complications following the use of vaginal meshes in pelvic floor reconstructive surgery [32,33,34]. In 2016, the FDA reclassified transvaginal meshes used in the treatment of pelvic floor disorders from intermediate risk class (II) to high risk class (III) [34]. A very common complication of mesh placement is its exposure to the vaginal canal: in the case of surgeries performed in the anterior compartment, on average, in 10% of cases (1.4–19.0%), compared to procedures involving other compartments, on average, in 17% (3–36%) [35,36]. In this study, we took steps to compare laparoscopy with other methods of mesh implantation.

Looking at other studies evaluating laparoscopic mesh implantation for the treatment of POP, the sample size ranged from 80 to 120 patients [29,37,38]. A total of 81 patients took part in our study, of which as many as 81% of them underwent laparoscopic surgery at the doctor’s discretion. The most common disorder was lowering of the anterior vaginal wall/urinary bladder—cystocele (62 patients after laparoscopic surgery, 13 patients after other types of surgery). In most cases, the patients were implanted with a two-arm gynecological mesh (81% of all patients). The preoperative data obtained showed that the most common type of disorder diagnosed in the patients was a prolapse of the anterior vaginal wall/bladder (cystocele), which was diagnosed in 75 patients. Prolapse of the uterus (uterine prolapse) was diagnosed in 55 patients, prolapse of the posterior vaginal wall/rectum (rectocele) in 45 patients, and prolapse of the vaginal vault/urethra (ureterocele) was diagnosed in 6 patients. After preoperative consultations, the patients underwent surgical treatment of pelvic floor dysfunction with gynecological mesh implantation. G-Mesh^®^ Posterior 2 mesh (two-arm) was used in 68 patients, Anterior 4 mesh (four-arm) was used in two patients, and Posterior 6 mesh (six-arm) was used in 11 patients. Only the two-arm meshes were implanted using the Dubuisson method (laparoscopically), whereas all three types of meshes were implanted using other techniques. Published works include information on the occurrence of various intraoperative complications, such as ureteral obstruction, bladder damage, or heavy bleeding [39,40]. In this study, no adverse events related to the implant were recorded during the surgery, and only two patients experienced intraoperative complications. Comparing the duration of the surgery, it can be concluded that it was similar for the two methods of implantation (83 min in the case of laparoscopic surgery and 85 min in the case of transvaginal surgery).

G-Mesh^®^ is a non-resorbable, light polypropylene gynecological mesh with a macroporous structure, which is characterized by low surface mass and atraumatic edges. After analyzing early postoperative information, it can be stated that there were no adverse events up to 4 days post operation related to the implanted product during hospitalization. About 93% rated the comfort associated with the mesh as very good; 90% of patients rated the pain as 0 on the VAS scale. The remaining patients complained, among other things, about pain at the site of trocar insertion. When assessing the sense of function in the first days after surgery, about 96% of patients rated it as good/very good.

The Clinical Trial Plan assumed two follow-up visits. All patients showed up for their first visit, which took place, on average, 76 days after the procedure. This allowed for a full assessment of the patient’s condition. Some patient visits took place a week after the procedure; for other results, we waited much longer (a few months) due to patients not showing up for the visit. Conducting a follow-up visit after a longer period of time allowed for the observation of the impact of mesh implantation on the effectiveness of surgical treatment of genital lowering in the long term (even after 14 months). At the first follow-up visits, no adverse events directly related to the use of the G-Mesh^®^ product were observed. Most patients reported no foreign body sensation (approx. 81%) and no postoperative pain (approx. 90%). However, approximately 59% of patients experienced postoperative complications, such as pulling in the lower abdomen and recurrent rectocele.

Only 42 patients showed up for the second, optional follow-up visit, and the visit took place on average 178 days after the procedure. Only two patients experienced an adverse event, which was a recurrent lowering of the reproductive organ, of which one patient required additional SSLF (Richter) surgery. Post-operative complications were noted in 38% of patients and mainly included recurrent rectocele. Considering the literature reports on bladder damage, we paid attention to the occurrence of stress urinary incontinence [39,40,41,42]. In the medical history, we noted SUI in seven patients. However, we did not notice the effect of POP surgery on the deterioration of bladder function, because at the second follow-up visit only five patients reported the occurrence of SUI. Foreign body sensation was reported by only 24% of patients. When assessing the average pain sensation using the VAS scale, 83% of patients reported no pain. This translated into high functional scores, as 88% of patients rated the function as good or very good. Comfort was also rated very high, as 88% of patients rated comfort as very good at the second follow-up visit.

By summarizing and analyzing the data collected during all follow-up visits, the effectiveness and safety of the G-Mesh^®^ implant can be assessed as effective and safe. An additional interview conducted with all patients an average of 318 days after the procedure confirmed the patients’ satisfactory feelings in terms of comfort and mean pain sensation. Patients rated comfort as very good or good in 90% of cases, while pain was rated as no pain (0) in 89%. The results from the interview are similar to previous assessments from follow-up visits, indicating a high level of patient satisfaction, minimal discomfort, and no significant pain. The evaluation of comfort and pain by patients after the procedure emphasizes that the G-Mesh^®^ implant is not only effective and safe but also imperceptible to patients, which translates into their high level of satisfaction and improved quality of life.

## 5. Conclusions

G-Mesh^®^ gynecological mesh implantation can be assessed as an effective and safe method in the treatment of pelvic floor dysfunction in women, both in the case of diagnosed cystocele, rectocele, uterine prolapse, and ureterocele. G-Mesh^®^ gynecological mesh can be successfully implanted using three methods: laparoscopically using the Dubuisson method, transvaginally, or using the Richardson method (laparotomy). Based on the obtained results, it can be stated that the implantation methods used are effective. However, due to the great popularity of laparoscopic methods, which are less invasive compared to other types of surgery, the availability of results for other techniques is limited. Considering the fact that a significant part of the problems described in the literature related to the use of mesh implants for supplying pelvic floor disorders occurs within a period of several years after the implantation, it is planned to conduct similar studies in the long-term period of 3–5 years after the surgery.

## Figures and Tables

**Table 1:** Types of meshes depending on the span, length, and width of arms.

Mesh Type	Arm Span (mm)	Arm Length	Arm Width
Posterior 2 (old version)	574	-	32
Posterior 2 (new version)	574	262	32
Anterior 4	418	174	15
Posterior 4	600	266 (lateral arm)	17

**Table 2:** Demographic data.

	Patients with Laparoscopic Procedures (*n* = 66)	Patients with Other Surgeries (*n* = 15)	*p* Values	All Patients (*n* = 81)
Age in years (range)	62 (28–81)	64 (38–83)	0.2643	62 (28–83)
BMI	25.53 (19.33–38)	25.61 (17.97–33.46)	0.2563	26 (18–38)
Medical history			0.9219	
Thyroid diseases	17 (25.8%)	4 (26.7%)		21 (25.9%)
Cardiovascular diseases	2 (3.0%)	2 (13.3%)		4 (4.9%)
Urinary diseases	6 (9.1%)	2 (13.3%)		8 (9.9%)
Digestive system diseases	7 (10.6%)	1 (6.7%)		8 (9.9%)
Respiratory system diseases	3 (4.5%)	0		3 (3.7%)
Osteoarthritis	6 (9.1%)	0		6 (7.4%)
Cerebral diseases	1 (1.5%)	0		1 (1.2%)
Skin diseases	1 (1.5%)	0		1 (1.2%)
Vascular diseases	7 (10.6%)	2 (13.3%)		9 (11.1%)
Bone diseases	2 (3.0%)	1 (6.7%)		3 (3.7%)
Diseases of the reproductive system	7 (10.6%)	3 (20.0%)		10 (12.3%)
Neurological disorders	2 (3.0%)	1 (6.7%)		3 (3.7%)
Diseases of the visual system	2 (3.0%)	2 (13.3%)		4 (4.9%)
Metabolic diseases	39 (59.1%)	11 (73.3%)		50 (61.7%)
Hernia surgery	1 (1.5%)	0		1 (1.2%)
Clinical condition	1 (1.5%)	0		1 (1.2%)
Mental disorders	2 (3.0%)	0		2 (2.5%)
Stress urinary incontinence (SUI)	5 (7.4%)	2 (15.4%)		7 (8.6%)
Overactive bladder (OAB)	4 (5.9%)	0		4 (4.9%)
None	14 (20.6%)	1 (7.7%)		15 (18.5%)
Medication intake			1.0000	
yes	48 (70.6%)	11 (84.6%)		60 (74.1%)
no	20 (29.4%)	1 (7.7%)		21 (25.9%)

**Table 3:** Preoperative data.

	Patients with Laparoscopic Procedures (*n* = 66)	Patients with Other Surgeries (*n* = 15)	*p* Values	All Patients (*n* = 81)
Type of disorder			0.0113	
lowering of the anterior vaginal wall/urinary bladder (Cystocele)	62 (93.9%)	13 (86.7%)		75 (92.6%)
lowering or prolapse of the uterus (Uterine prolapse)	51 (77.3%)	4 (26.7%)		55 (67.9%)
lowering of the vaginal vault/urethra (Ureterocele)	5 (7.6%)	1 (6.7%)		6 (7.4%)
lowering of the posterior vaginal/rectal wall (Rectocele)	41 (62.1%)	4 (26.7%)		45 (55.6%)
lowering of the uterorectal cavity/small intestine (Enterocele)	0	0		0
Gynecological examination			0.0297	
Post-hysterectomy (including/excluding appendages)	0	7 (46.7%)		7 (8.6%)
Post-Dubuisson surgery	0	1 (6.7%)		1 (1.2%)
Post- anterior/posterior surgery	0	3 (20%)		3 (3.7%)
Urogynecologycal examination			0.0468	
Bladder POPQ into front vaginal wall				
level I	5(33.3%)	0		5 (6.2%)
level II	20 (30.3%)	4 (26.7%)		24(29.6%)
level III	25 (37.9%)	7 (46.7%)		32 (39.5%)
level IV	11 (16.7%)	3 (20.0%)		14 (17.3%)
Rectum POPQ into back vaginal wall				
level 0	15 (22.7%)	5(33.3%)		20(24.7%)
level II	21(31.8%)	4 (26.7%)		25(30.9%)
level III	11 (16.7%)	1 (6.7%)		12 (14.8%)
level IV	3 (4.5%)	0		3 (3.7%)
Cervix POPQ				
level 0	7 (10.6%)	3(20%)		10 (12.3%)
level II	18 (27.3%)	2 (13.3%)		20 (24.7%)
level III	17 (25.8%)	1 (6.7%)		18 (22.2%)
level IV	13 (19.7%)	0		13 (16.0%)
Cough test			0.0039	
negative	2 (3.0%)	3 (20.0%)		5 (6.2%)
no data	64 (97%)	12 (80.0%)		76 (93.8%)

**Table 4:** Information regarding surgeries.

	Patients with Laparoscopic Procedures (*n* = 66)	Patients with Other Surgeries (*n* = 15)	*p* Values	All Patients (*n* = 81)
Type of products used			0.0572	
Posterior 2 old	3 (4.5%)	2 (13.3%)		5 (6.2%)
Posterior 2 new	63 (95.5%)	0		63 (77.8%)
Anterior 4	0	2 (13.3%)		2 (2.5%)
Posterior 6	0	11 (73.3%)		11 (13.6%)
Surgery time in minutes (range)	83 (50–201)	85 (70–190)	0.0010	83 (50–201)
Perioperative complications			1.0000	
tightening of the postoperative wound on the right side	2 (3.0%)	0		2 (2.5%)
problems with dissecting the bladder from the vagina–scars after anterior surgery	1 (1.5%)	0		1 (1.2%)
Implant-related adverse events	0	0	-	0

**Table 5:** Early post-operative information.

	Patients with Laparoscopic Procedures (*n* = 66)	Patients with Other Surgeries (*n* = 15)	*p* Values	All Patients (*n* = 81)
Mean hospitalization time in days (range)	3 (1–4)	3 (3–5)	0.0014	3 (1–5)
Use of antibiotics			1.0000	
Yes	55 (83.3%)	13 (86.7%)		68 (84.0%)
No	11 (16.7%)	2 (13.3%)		13 (16.0%)
Complications in first days post surgery			0.0000	
recurring cystocele/rectocele	1 (1.5%)	0		1 (1.2%)
pain at the laparoscopic puncture site	1 (1.5%)	0		1 (1.2%)
Adverse events related to the implant	0	0	-	0
Mesh-related comfort/discomfort assessment			0.0000	
very good	65 (98.5%)	14 (93.3%)		79 (98.0%)
good	1 (1.5%)	0		1 (1.2%)
no data	0	1 (6.7%)		1 (1.2%)
Mean pain assessment VAS (1–10)			0.0000	
0	66 (100%)	13 (86.7%)		79 (97.5%)
2	2 (3.0%)	1 (6.7%)		3 (3.7%)
3	0	1 (6.7%)		1 (1.2%)
Function/disfunction assessment			0.0000	
very good	2 (3.0%)	1 (6.7%)		3 (3.7%)
good	64 (97.0%)	11 (73.3%)		75 (92.6%)
no data	2 (3.0%)	1 (6.7%)		3 (3.7%)

**Table 6:** Follow-up outcomes (first control visit).

	Patients with Laparoscopic Procedures (*n* = 66)	Patients with Other Surgeries (*n* = 15)	*p* Values	All Patients (*n* = 81)
Follow up in days (range)	79 (22–429)	63 (6–181)	0.0118	76 (6–429)
Complications			1.0000	
related to urinary tract	17 (25.8%)	1 (6.7%)		18 (22.2%)
post-operative pain	26 (39.4%)	1 (6.7%)		27 (33.3%)
de novo pelvic organ prolapse	23 (34.8%)	0		23 (28.4%)
constipation	4 (6.1%)	0		4 (4.9%)
recurrent vaginal infections	1 (1.5%)	0		1 (1.2%)
difficulties in passing stool	2 (3.0%)	0		2 (2.5%)
wound infection	1 (1.5%)	0		1 (1.2%)
discomfort in rectal region	0	1 (6.7%)		1 (1.2%)
qualification for TOT surgery	0	1 (6.7%)		1 (1.2%)
Adverse events	0	0	-	0
Effectiveness			0.0000	
very good	60 (90.9%)	15 (100%)		75 (92.6%)
good	3 (4.5%)	0		3 (3.7%)
poor	2 (3.0%)	0		2 (2.5%)
no data	1 (1.5%)	0		1 (1.2%)
Comfort			0.0000	
very good	60 (90.9%)	15 (100%)		75 (92.6%)
good	2 (3.0%)	0		2 (2.5%)
poor	3 (4.5%)	0		3 (3.7%)
no data	1 (1.5%)	0		1 (1.2%)
Mean pain sensation VAS (1–10)			0.0006	
0	58 (87.5%)	15 (100%)		73 (90.1%)
2	4 (6.1%)	0		4 (4.9%)
4	1 (1.5%)	0		1 (1.2%)
5	3 (3.0%)	0		3 (3.7%)
Function/dysfunction assessment			0.0000	
very good	8 (12.1%)	1 (6.7%)		9 (11.1%)
good	56 (84.8%)	13 (86.7%)		69 (85.2%)
poor	2 (3.0%)	0		2 (2.5%)
no data	0	1 (6.7%)		1 (1.2%)
Foreign body sensation			0.3148	
yes	7 (10.6%)	1 (6.7%)		8 (9.9%)
no	54 (81.8%)	12 (80.0%)		66 (81.5%)
no data	5 (7.6%)	2 (13.3%)		7 (8.6%)

**Table 7:** Follow-up outcomes (second follow-up visit).

	Patients with Laparoscopic Procedures (*n* = 38)	Patients with Other Surgeries (*n* = 4)	*p* Values	All Patients (*n* = 42)
Follow up in days (range)	175 (49–429)	213 (81–336)	0.0000	178 (49–429)
Complications			1.0000	
post TOT-surgery	0	1 (25%)		1 (2.4%)
overactive bladder	1 (2.6%)	1 (25%)		2 (4.8%)
vaginal itchiness	1 (2.6%)	0		1 (2.4%)
chronic postoperative pain—lower abdominal pain	6 (15.8%)	0		6 (14.3%)
stress urinary incontinence	5 (13.2%)	0		5 (11.9%)
pelvic organ prolapse	3 (7.9%)	0		3 (7.1%)
chronic pain at implantation site	1 (2.6%)	0		1 (2.4%)
constipation	3 (7.9%)	0		3 (7.1%)
feeling of incomplete emptying of the bladder	1 (2.6%)	0		1 (2.4%)
pulling sensation at passing stool	1 (2.6%)	0		1 (2.4%)
recurrent rectocele	12 (31.6%)	0		12 (28.6%)
recurrent cystocele	4 (10.5%)	0		4 (9.5%)
cervix elongation	1 (2.6%)	0		1 (2.4%)
pulling sensation in bladder area	1 (2.6%)	0		1 (2.4%)
nycturia	1 (2.6%)	0		1 (2.4%)
pelvic heaviness	1 (2.6%)	0		1 (2.4%)
bulging in vagina	3 (7.9%)	0		3 (7.1%)
fecal incontinence	1 (2.6%)	0		1 (2.4%)
micturition disorder	2 (5.3%)	0		2 (4.8%)
dyspareunia	1 (2.6%)	0		1 (2.4%)
vaginal dryness	1 (2.6%)	0		1 (2.4%)
fungal infection	1 (2.6%)	0		1 (2.4%)
recurrent enterocele	1 (2.6%)	0		1 (2.4%)
symptomatic pelvic organ prolapse	1 (2.6%)	0		1 (2.4%)
pulling sensation at implantation site	1 (2.6%)	0		1 (2.4%)
total vaginal and uterine prolapse	1 (2.6%)	0		1 (2.4%)
none	16 (38.1%)			16 (38.1%)
Adverse events	2 (5.3%)	0	-	2 (4.8%)
none	39 (92.9%)			39 (92.9%)
no data	1 (2.4%)			1 (2.4%)
Efficiency			0.0324	
very good	26 (68.4%)	4 (100%)		30 (71.4%)
good	8 (21.1%)	0		8 (19.0%)
poor	3 (7.9%)	0		3 (7.1%)
no data	1 (2.6%)	0		1 (2.4%)
Comfort			0.0354	
very good	33 (86.8%)	4 (100%)		37 (88.1%)
good	0	0		0
poor	4 (10.5%)	0		4 (9.5%)
no data	1 (2.6%)	0		1 (2.4%)
Mean pain sensation VAS (1–10)			0.000	
0	31 (81.6%)	4 (100%)		35 (83.3%)
2	2 (5.3%)	0		2 (4.8%)
3	1 (2.6%)	0		1 (2.4%)
4	1 (2.6%)	0		1 (2.4%)
5	2 (5.3%)	0		2 (4.8%)
no data	1 (2.6%)	0		1 (2.4%)
Function/dysfunction assessment			0.0018	
very good	8 (21.1%)	2 (50%)		10 (23.8%)
good	26 (68.4%)	1 (25%)		27 (64.3%)
poor	4 (10.5%)	0		4 (9.5%)
no data	1 (2.6%)	0		1 (2.4%)
Foreign body sensation			1.0000	
yes	10 (26.3%)	0		10 (23.8%)
no	27 (71.1%)	2 (50%)		29 (69.0%)
no data	1 (2.6%)	2 (50%)		3 (7.1%)

**Table 8:** Follow-up outcomes (third follow-up visit—interview)

	Patients with Laparoscopic Procedures (*n* = 66)	Patients with Other Surgeries (*n* = 15)	*p* Values	All Patients (*n* = 81)
Follow up in days (range)	321 (135–1430)	440 (138–1430)	0.0000	318 (135–1430)
Comfort			0.0587	
very good	56 (84.8%)	12 (80.0%)		68 (84.0%)
good	2 (3.0%)	3 (20.0%)		5 (6.2%)
poor	3 (4.5%)	0		3 (3.7%)
no data	5 (7.6%)	0		5 (6.2%)
Mean pain sensation VAS (1–10)			0.0000	
no data	1 (1.5%)	0		1 (1.2%)
0	57 (86.4%)	15 (100%)		72 (88.9%)
2	1 (1.5%)	0		1 (1.2%)
3	2 (3.0%)	0		2 (2.5%)
4	2 (3.0%)	0		2 (2.5%)
5	1 (1.5%)	0		1 (1.2%)

## Data Availability

The data presented in this study are available upon request from the corresponding author.
